# A Protest

**Published:** 1896-09

**Authors:** 


*Editors Texas Medical Journal:*

### A Protest.

The following letter from a well educated young physician will be read with interest. It speaks volumes against the proposed degradation of medicine. Fancy the fiery zeal and ambition that must nerve a young student studying medicine to know that when he graduates and goes home with his diploma, perhaps granted after a most searching examination by one of the ablest college faculties in America, he will have to submit to the *humiliation*, as Dr. Kenney did, of being examined by a mixed board—by a homeopath, and, perhaps, by an eclectic, parties who do not pretend ever to have studied medicine, and, therefore, are not expected to know anything about our system of practice. Here is the letter:

SAN ANTONIO, TEXAS, August 11, 1896.

*Editors Texas Medical Journal:*

I desire to place myself on record as opposed to the State Medical Association asking for a mixed board, and I avail myself of the opportunity to do so through the columns of the TEXAS MEDICAL JOURNAL.

The regular profession does not recognize any sect in the practice of medicine, and, therefore, should not become contaminated by mixing with "paths" and "isms" in any way. It is certainly humiliating for a young man to return home after receiving a regular medical education, and have to submit to an examination by the representative of some "sect." It was not only humiliating in my case, but likewise ridiculous.

With kind regards, very truly yours,

JNO. W. KENNEY, M. D.

